# Neonatal Heart Responds to Pressure Overload With Differential Alterations in Various Cardiomyocyte Maturation Programs That Accommodate Simultaneous Hypertrophy and Hyperplasia

**DOI:** 10.3389/fcell.2020.596960

**Published:** 2020-11-19

**Authors:** Xiaoning Ding, Shoubao Wang, Ye Wang, Junjie Yang, Nan Bao, Jinfen Liu, Zhen Zhang

**Affiliations:** ^1^Shanghai Children’s Medical Center, Pediatric Translational Medicine Institute and Shanghai Pediatric Congenital Heart Disease Institute, Shanghai Jiao Tong University School of Medicine, Shanghai, China; ^2^Department of Thoracic and Cardiovascular Surgery, Shanghai Children’s Medical Center, Shanghai Jiao Tong University School of Medicine, Shanghai, China; ^3^Department of Pediatric Surgery, Shanghai Children’s Medical Center, Shanghai Jiao Tong University School of Medicine, Shanghai, China

**Keywords:** cardiomyocyte maturation, pressure overload, pulmonary artery banding, neonatal heart, Metabolic transition

## Abstract

Pressure overload is one of the pathophysiological conditions commonly associated with right-sided congenital heart disease (CHD). Patients suffer from this condition right after birth. However, little is known about how neonatal heart reacts to it. We have previously established a pulmonary artery banding (PAB) model in neonatal rat. Here we show that PAB accelerated transition of mononuclear cardiomyocytes into multinucleated cells to promote hypertrophic growth in neonatal heart. The elevated afterload significantly increased the mitotic activities of neonatal cardiomyocytes. Consistent with the proliferative potential, the elevated pressure overload also increased cytokinetic marker counts of cardiomyocytes. Using cardiomyocyte-specific lineage tracing, we noticed a clonal expansion of rare unlabeled cardiomyocytes in the PAB group, revealing a subgroup of cardiomyocytes with a strong capability of proliferation. In addition, PAB hearts at post-banding day 7 didn’t have the accumulation of macrophages, which is an immune response essential for neonatal heart regeneration in injury models. Transcriptomic analyses revealed that neonatal PAB induced an expression profile featuring both cardiomyocyte hypertrophy, such as highly activated translation, oxidative phosphorylation, and mitochondrial biogenesis programs etc., and immature cardiomyocyte, such as enhanced cell cycle activities and glycolytic metabolism, down-regulated cytoskeleton and ion channel gene expression, and maintenance of fetal-specific sarcomeric isoforms etc. It indicates that pressure overload has differential impacts on various cardiomyocyte maturation (CM) programs that may contribute to the concurrent cardiomyocyte hypertrophy and hyperplasia. The bivalent status of transcriptional profile highlights the plasticity of neonatal cardiomyocytes that can be exploited to adapt the postnatal environment.

## Introduction

Mammalian circulatory system undergoes dramatic changes after birth to adapt to postnatal life (Hew and Keller, 2003). Particularly, the inflation of lungs establishes the pulmonary circulation and the closure of prenatal shunts separates the pulmonary circulation from systemic circulation. These circulatory alterations switch the gas exchange from the placenta to the lungs and shift two ventricles to function in tandem instead of in parallel. Meanwhile, neonatal hearts go through a critical period of biochemical and mechanical maturation to cope with the extrauterine life (Kannan and Kwon, 2020). This neonatal transition is particularly sensitive to perturbation. The problem of neonatal transition can render individual susceptible to adult cardiac disease (Anatskaya et al., 2010).

Congenital heart diseases (CHD) cause a number of morphological defects that change the circulatory hemodynamics. For example, pressure overload of right ventricle (RV) is a common pathophysiological condition associated with CHD, such as RV outflow obstruction and systemic RV etc. These pathological conditions usually exist at birth and may infringe upon the postnatal growth. For optimal clinical care of these patients, it is crucial to understand how the neonatal myocardium responds to the pathological conditions during the complex transition stage. However, our understandings of the adaptive physiology under these pathological conditions is limited due to the lack of appropriate neonatal models.

We have previously generated the first neonatal right ventricle overload model by constricting pulmonary artery within 6 h after birth in rat and the banding causes significantly enlarged right ventricle at postnatal day 7(4). Here we used this model to evaluate how neonatal myocardium adapts to increased afterload. Similar to the adult hearts, neonatal cardiomyocytes presented hypertrophic growth in response to pressure overload. On the other hand, neonatal hearts also had significantly increased numbers of mitotic and cytokinetic markers in cardiomyocytes, indicating an increased cardiomyocyte proliferation. To validate this observation, we established the PAB protocol in neonatal mice to take advantage of available cell lineage-tracing tools in mouse. Using inducible cardiomyocyte-specific *Tnn2-rtTA; TetO-Cre* and *Rosa26^RFP/+^* reporter, we detected the clonal expansion of rare RFP^–^ cardiomyocytes. Although we found a significantly increased cardiomyocyte proliferation, there was no accumulation of pro-proliferative M2 macrophages in post-banding day 7 (P7) PAB hearts. It indicates that the proliferative capability is more of an intrinsic property of neonatal cardiomyocytes. Using RNA-seq analysis, we revealed a unique expression profile with diverged CM program responses that may contribute to the seemingly contradictory cardiomyocyte behaviors.

## Materials and Methods

### Animals and Surgery

Surgical protocols used in this investigation were approved by the Animal Care and Use Committee of Shanghai Children’s Medical Center. PAB or sham operations were performed on Sprague-Dawley rats or C57Bl/6J mice within 6 h after birth and in adherence to the guidelines for the Care and Use of Laboratory Animals.

Briefly, neonates were anaesthetized by hypothermia. It led to asystole and reversible apnea and prevented excessive blood loss. Next, they were transferred to ice bed and fixed in the supine position under a stereomicroscope. Following a transverse skin incision, horizontal thoracotomy was performed by dissecting intercostal muscles and cutting the sternum. The incision site was at the second intercostal space for mice and the third intercostal space for rats. After opening the pericardium, we carefully separated the pulmonary artery from the aorta under microscope. A 11-0 nylon thread was positioned under pulmonary artery and a 30G(rat)or 31G (mice) needle was placed upon the pulmonary artery. The pulmonary artery and needle were then tied together by the thread. After the needle was quickly removed, a fixed constricted opening equal to the diameter of the needle was created in the lumen of pulmonary artery. The sternum and thoracic wall were sutured with a 9-0 (rat) or 10-0 (mouse) nylon thread. Afterward, neonates were removed from ice bed and placed under a heat plate to warm up for several minutes until natural movements and a red/pink complexion were achieved. Neonates were then returned to their mother. The sham group underwent the same procedure except the banding step.

The following mouse lines have been described previously: *Tnn2-rtTA; TetO-Cre* (Wu et al., 2010), *Rosa26^*RFP*^* (Madisen et al., 2010). All the lines were maintained in C57Bl/6J background. For lineage tracing experiment, 0.3 ml doxycycline (12 mg/ml, Beyotime ST039A) was applied through oral gavage at E17.5 and E18.5.

### Immunofluorescent Staining

Hearts were embedded in O.C.T. compound (Tissue-Tek, Sakura, NL) and cryosectioned at 4.5μm. Immunofluorescent staining was performed according to standard procedure with antibodies against phosphorylated histone H3 (PH3 ab47297, Abcam), Ki67 (ab16667, Abcam) and Cardiac Troponin T (cTnT) (ab125266, ab8295, Abcam). Alexa fluor 488- and 555- conjugated antibodies (A11011 and A21428, Invitrogen, Life Technologies) were used as secondary antibodies. DAPI (D1306 Invitrogen, Life Technologies) was used for nuclei staining. In addition, Alexa Fluor^®^ 488 Conjugate of wheat germ agglutinin (WGA) (W11261, Invitrogen, Life Technologies)was used to visualize cardiomyocyte membrane. Microscopic examinations were performed using a laser confocal microscope Leica TCS SP8.

### Counting of Cardiomyocyte Nucleus Number

Cardiomyocytes from right ventricular of 3-day-old and 7-day-old Sprague Dawley rats were isolated as previously described (Wang et al., 2016). Dissociated cells were fixed with Click-iT^TM^ fixative (C10632 Invitrogen, Life Technologies) at room temperature for 30 min, washed with phosphate-buffered saline (PBS) and then smeared on slide glasses. Cardiomyocytes were visualized by cardiac cTnT staining (ab125266, Abcam) and DAPI. Images were photographed under Leica TCS SP8 and nucleus number per cardiomyocyte was recorded.

### Ploidy Analysis of Cardiomyocytes

DNA content measurements by FACS were performed as described previously (Rabinovitch, 1983). Briefly, Isolated cells were fixed and permeabilized with BD Cytofix/CytopermTM solution (BD 554714) and stained with cardiac anti-cTnT antibody (ab125266, Abcam) and Propidium Iodide (P3566 Invitrogen, Life Technologies). Samples were analyzed on a BD FACSAria cell sorter (BD Biosciences, San Jose, CA, United States). Ploidy of cardiomyocyte was assessed in Flowjo, using at least 10,000 cardiomyocytes for each sample.

### TUNEL Staining

Apoptosis was examined by TdT mediated FITC-12-dUTP Labeling (TUNEL) kit (Yeasen, T18120). In brief, cryosections were treated with proteinase K (20 μg/ml) and then with TdT incubation buffer in the presence of FITC-12-dUTP Labeling mix and TdT enzyme at 37°C for 1 h. Positive control was treated by DNase I (1 μg/ml) before incubating with TdT buffer. ddH_2_O instead of TdT enzyme was used in negative control. DAPI (D1306, ILife Technologies) was used for nuclei staining.

### Flow Cytometry

Mouse cardiac ventricles were harvested and minced into ∼1 mm^3^ small pieces. Samples were digested with 0.1% Collagenase (Gibco, #17101-015) with 2.5 U/ml Dispase II (Sigma, #D4693). Cell suspension was filtered through 70 μm cell strainer. Cells were incubated with antibodies for 1 h at 4°C prior to flow cytometry analysis. The following antibodies were used: APC Rat anti mouse CD45 (BD Pharmingen, 559864, 1:200), FITC-conjugated anti-CD11b antibody (BD Pharmingen, 557396), PE-conjugated anti-F4/80 antibody (eBioscience, 12-4801-82), and PE-CyTM7-conjugated anti-Ly6c antibody (BD Pharmingen, 560593). All FACS gates for fluorophore-labeled monoclonal antibodies were defined using appropriate isotype controls. Due to the small number of immune cells in one heart, 3–4 hearts were pooled together as a single sample. The total cell number was normalized to heart weight for comparison. Data analysis was performed in Flowjo software.

### Q-PCR

Total RNA was extracted from P7 rat ventricles using TRIzol^®^ LS Reagent (10296028, Thermo Fisher Scientific). Total RNA was reversely transcribed with Prime Script^TM^ RT reagent Kit (RR037A, TaKaRa) to obtain cDNA. Quantitative real-time PCR was performed with SYBR Green method on a QuantStudio^TM^ 7 Flex Real-time PCR system (ThermoFisher Scientific). Primers for each gene are list as the following:

Bnp: 5′-CCTCTTCCTGGCCTTTTGG-3′, 5′-TGTGTTGG ACACCGCACTGT-3′;
Anp: 5′-GCTGCAGACTCCGGCTTCT-3′, 5′-ATCACTTGA GAGGTGGTCCCA-3′;
Gapdh: 5′-CCTTCATTGACCTCAACTAC-3′; 5′-GGAAGGC CATGCCAGTGAGC-3′

### RNA-Seq and Bioinformatic Analysis

Total RNA was extracted from P3 and P7 mouse ventricles using TRIzol^®^ LS Reagent (10296028, Thermo Fisher scientific). Library preparation and transcriptome sequencing on an HiSeq X PE150 platform were performed through a commercial service provided by Mingma Technologies Co., Ltd. (Shanghai, China). to generate 100 bp paired-end reads.

For bioinformatic analysis, we used STAR (version 2.5.2b) to align raw reads to mouse genome (Gencode, GRCm38). We use R package DESeq2 (version 1.20.0) (Love et al., 2014) to identify differential expressed genes, with threshold of fold change ≥ 1.5 and *p* ≤ 0.05. Differential expressed genes were submitted to Metascape^1^ for gene ontology analysis (Zhou et al., 2019). To evaluate average gene expression of each functional categories, we took mean of normalized gene expression value of each gene in the functional category and plotted with logarithmic transformation. We used R package pheatmap to plot heatmap of gene expression.

### Quantification and Statistical Analysis

The number of proliferating cardiomyocytes was assessed by counting Ki67^+^/cTnT^+^ or PH3^+^/cTnT^+^ cells in right ventricle. Three histological sections from comparable positions of each heart were used for quantification. Six hearts were included in each group. Three hearts per group were applied for lineage-tracing study. Six sections from comparable positions of each heart were used for quantification. Cardiomyocyte size was quantified by measuring the cross sectional area along the short axis of cardiomyocytes triple-stained by WGA (green), anti-cTnT (red), and DAPI (blue). About 200 cells from each heart were assessed by Image J. Six hearts were used for each group. Quantitative data are presented as Mean ± SEM. Statistical analyses were performed by two-tailed Student’s *t*-test. *p* < 0.05 is considered significant.

## Results

### PAB Induced Cardiomyocyte Hypertrophy in Neonates

**FIGURE 1:**
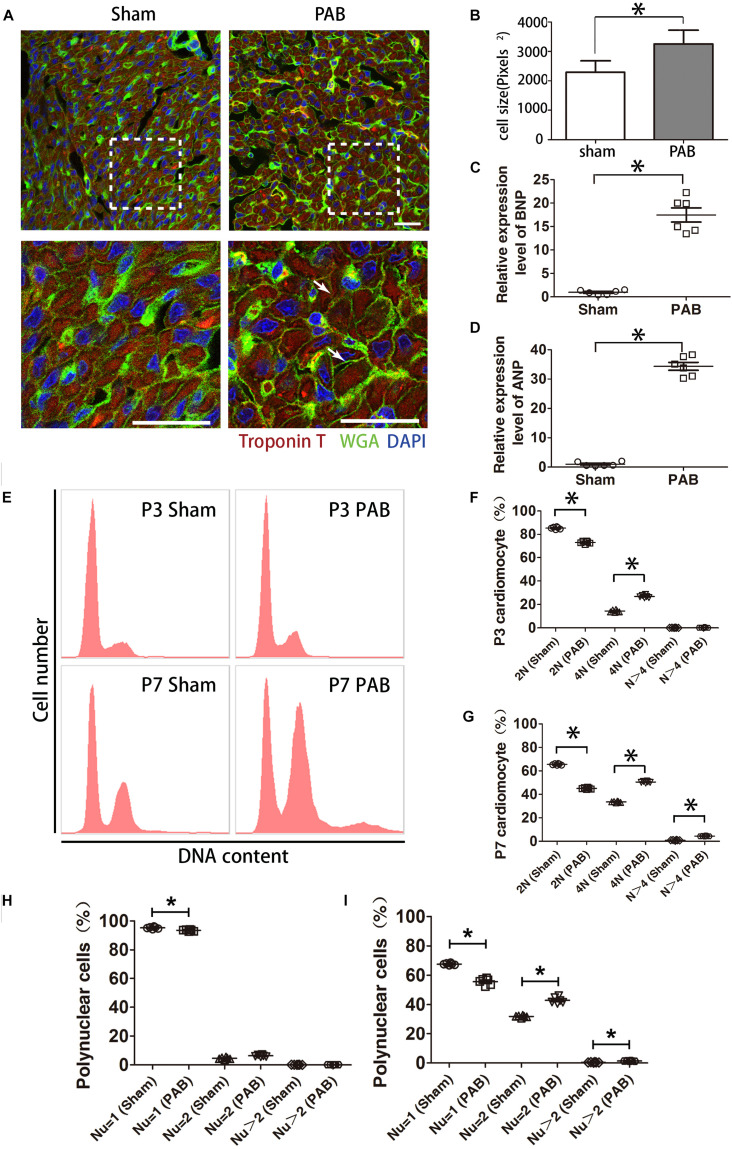
Cardiomyocyte hypertrophy in the first postnatal week PAB heart. **(A)** Immunofluorescent staining on heart sections from P7 heart. WGA, green. cTnT, red. DAPI, Blue. Arrows, hypertrophic cardiomyocytes. **(B)** Quantification of cardiomyocyte size (*n* = 6). **(C,D)** qPCR analysis of cardiac hypertrophy markers ANP and BNP (*n* = 6). **(E)** Representative FACS result of polyploidy analysis. **(F,G)** Comparison of polyploidy pattern between the sham and PAB groups at P3 **(F)** and P7 **(G)** (*n* = 6 for each time point). **(H,I)** Percentages of mono-, bi- and multinucleated cardiomyocytes in P3(H) and P7 (I) (*n* = 6 for each time point). Nu, nuclear number. Data are presented as Mean ± SEM. Statistic analysis was performed with two-tailed Student’s *t*-test, *, *p* < 0.05. Scale bar: 25 μm in low magnification panel and 6.25 μm in high magnification panel **(A)**.

We previously established a neonatal PAB rat model that quickly induces significantly thickened RV wall (Wang et al., 2017). It is well known that myocardium reacts to pressure overload through hypertrophy at adult stage (Pitoulis and Terracciano, 2020), we wondered whether cardiomyocyte hypertrophy is also involved in PAB-induced RV enlargement at the neonatal stage. The size of cardiomyocytes was measured by fluorescence-conjugated wheat germ agglutinin (WGA) staining (Figure 1A). WGA binds to cytoplasmic membrane protein and outlines cell shape. Results indicated that the average cardiomyocyte size increased about 42% at post-surgery day 7 (P7) after PAB (Figure 1B). The expression levels of two cardiac hypertrophy markers, atrial natriuretic peptide (ANP) and brain natriuretic peptide (BNP), also significantly increased by 33.3- and 16.5-fold, respectively (Figures 1C,D). These data indicate that cardiomyocyte hypertrophy is also a critical remodeling process responding to PAB in neonates.

Polyploidization is often a cellular response associated with cardiomyocyte hypertrophy. To this end, we measured DNA content with flow cytometry. Data showed that the number of diploid cardiomyocytes significantly reduced and tetraploid cardiomyocytes significantly increased in the PAB group at P3 (Figures 1E,F). At P7, the percentage of diploid cardiomyocytes continued to decrease while the percentage of tetraploid cardiomyocytes further increased to the extent beyond the percentage of diploid cardiomyocytes (Figures 1E,G). The number of polyploid cardiomyocytes (*N* > 4) was also significantly higher in the PAB group (Figure 1G).

The characteristic growth mode of rodent neonatal cardiomyocyte is to transit from proliferation to hypertrophy through cardiomyocyte multinucleation concomitant with increased cell volume. On the basis of physiological multinucleation, we found that PAB could accelerate this process. The percentage of mononuclear cardiomyocytes was significantly lower while the percentage of binuclear cardiomyocytes significantly higher in the PAB group at P3 (Figure 1H). These differences were further broadened at P7 and the percentage of multinuclear cells (Nu > 2) in the PAB group became significantly higher at this time point (Figure 1I). Since pressure overload-induced hypertrophy is accompanied by increased cell death in adult model (van der Bruggen et al., 2017), we evaluated cell apoptosis in our neonatal PAB hearts. Result indicated that there was no significant increase of apoptotic cardiomyocytes in P7 PAB hearts (Supplementary Figure S1).

### Neonatal Cardiomyocytes Showed a Higher Level of Proliferative Activity Under Pressure Overload

**FIGURE 2:**
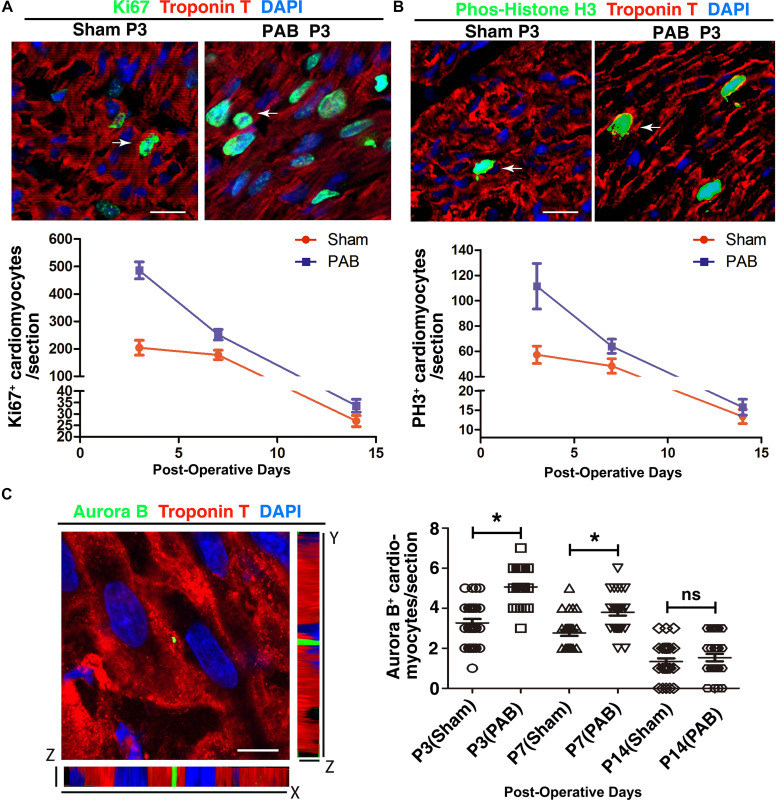
Cardiomyocyte proliferation in the sham and PAB hearts. **(A)** Representative immunofluorescent image for Ki67 (green), cTnT (red) and DAPI (blue) staining. Arrows, Ki67^+^ cardiomyocytes. Lower panel is the quantification of Ki67^+^ cardiomyocytes at P3, P7, and P14 (*n* = 6 for each time point). **(B)** Representative immunofluorescent image for PH3 (green), cTnT (red) and DAPI (blue) staining. Arrows, PH3^+^ cardiomyocytes. Lower panel is the quantification of PH3^+^ cardiomyocytes at P3, P7, and P14 (*n* = 6 for each time point). **(C)** Representative immunofluorescent image for Aurora B (green), cTnT (red), and DAPI (blue) staining. Right panel, quantification of Aurora B^+^ cardiomyocytes at P3, P7, and P14 (*n* = 6 for each time point). Data are presented as Mean ± SEM. Statistic analysis was performed by two-tailed Student’s *t*-test. *, *p* < 0.05. Scale bar: 12 μm in high magnification panels **(A,B)** and 5.5 μm **(C)**.

Neonatal heart has remarkable regenerative capability and can replace myocardial loss through cardiomyocyte proliferation (Lam and Sadek, 2018). Therefore, we examined whether PAB could induce enhanced cardiomyocytes proliferation. To detect proliferating cardiomyocytes, we co-stained sections with antibodies against cell mitotic proliferation marker Ki67/phosphorylated histone H3 (PH3) and cardiomyocyte-specific marker cTnT. Consistent with decreasing regenerative capability of myocardium (Porrello et al., 2011), the number of cardiomyocytes with positive mitotic markers was decreasing in both sham and PAB groups along the time (Figures 2A,B). But the number of mitotic marker-positive cardiomyocyte in the PAB group was higher than that of the sham group at all the time points. The percentage increase for Ki67^+^cTnT^+^ and PH3^+^cTnT^+^ cells were, respectively, 137.9% and 94.5% at P3, 41.4% and 32.0% at P7, 22% and 20.0% at P14 (Figures 2A,B).

Since postnatal cardiomyocytes undergo multinucleation, the increased levels of mitotic markers do not necessarily mean cell division. To verify whether some of the observed mitotic activities represent real cardiomyocyte proliferation, we used immunofluorescence staining to examine the expression of cytokinetic marker Aurora B kinase (AuroB) (Figure 2C). As expected, the number of AuroB^+^ cardiomyocytes significantly increased in both P3 and P7 PAB groups, again with more AuroB^+^ cardiomyocytes in the P3 group than in the P7 group. At P14, the number of AuroB + cardiomyocytes further decreased to nearly undetectable and no significant difference was detected between the sham and PAB groups. These data demonstrate that pressure overload resulting from PAB induces remarkable cardiomyocyte hyperplasia in neonates.

### Clonal Expansion of Cardiomyocytes in PAB Hearts

**FIGURE 3:**
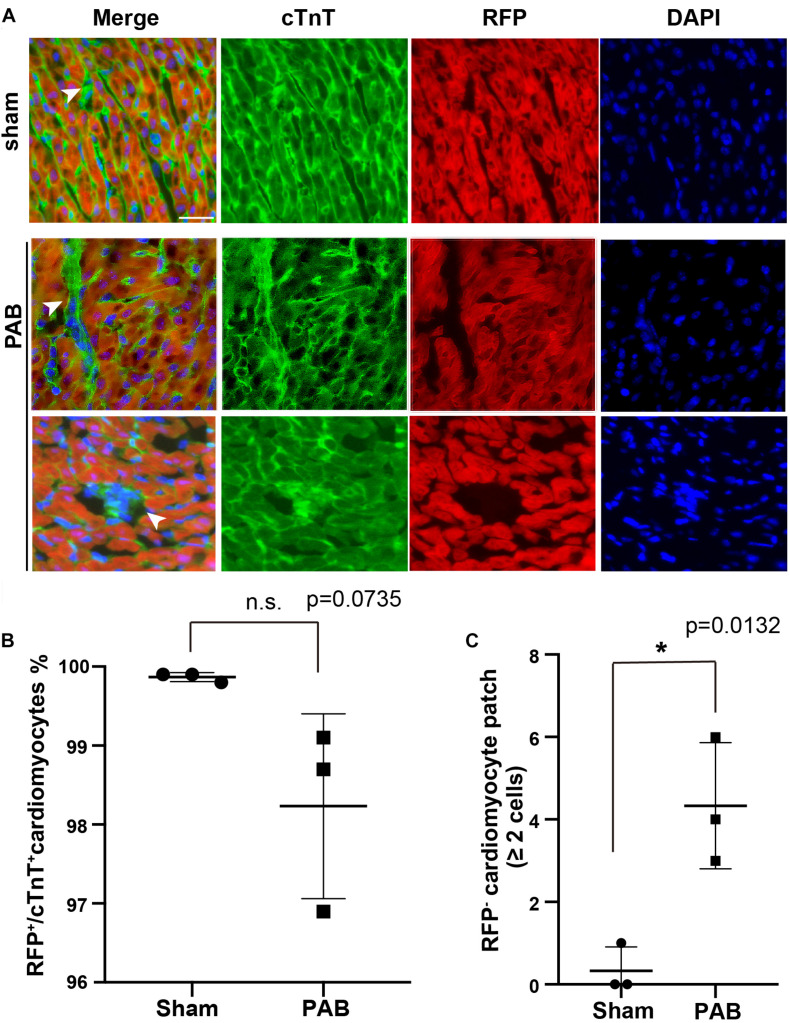
PAB induced clonal expansion of rare unlabeled cardiomyocytes in cardiac-specific lineage tracing. **(A)** Representative immunofluorescent image on P7 *Tnn2-rtTA; TetO-Cre*; *Rosa26^RFP/+^* heart sections stained with anti-cTnT antibody and DAPI. Green, cTnT; Red, RFP; Blue, DAPI. Arrowhead, cTnT^+^RFP^–^ cardiomyocytes. **(B)** Quantification of cTnT^+^RFP^+^ cardiomyocytes (*n* = 3). **(C)** Quantification of the number of cTnT^+^RFP^–^ cardiomyocyte clones with more than 2 cells (*n* = 3). Data are presented as Mean ± SEM. Statistic analysis was performed with two-tailed Student’s *t*-test. *, *p* < 0.05. Scale bar: 25 μm.

To visualize the pattern of cardiomyocyte proliferation in neonatal PAB model, we used a dual transgene tool of *Tnn2-rtTA; TetO-Cre*, together with a reporter *Rosa26^*RFP*^*, to perform lineage tracing in mice. We noted that a few of single scattered cardiomyocytes were not labeled by RFP after doxycycline treatment at E17.5 and E18.5 (Figure 3A). Since non-cardiomyocyte does not generate any cardiomyocyte beyond E11.5 (Li et al., 2019), these RFP^–^ cardiomyocytes are likely to be the cells escaped from lineage labeling rather than newly derived from cardiac progenitors after doxycycline treatment. The single scattered distribution of RFP^–^ cardiomyocytes renders it a good tool for clonal analysis.

E17.5-E18.5 *Tnn2-rtTA; TetO-Cre; Rosa26^*RFP/*+^* embryos were treated with doxycycline. Within 6 h after birth, we performed PAB on neonatal hearts and samples were collected at P7 for immunofluorescent staining. First, we examined the ratio of RFP^–^ cardiomyocytes in hearts. We noted that the ratio of RFP^+^/cTnt^+^ cardiomyocyte in the PAB group was slightly decreased in comparison to the sham group, although it was not statistically significant (Figure 3B). Unlike the single scattered RFP^–^ cardiomyocytes in the sham group, the majority of RFP^–^ cardiomyocytes were clustered together in the PAB group (Figure 3A), suggesting a clonal growth of cardiomyocyte. We barely saw RFP^–^ cardiomyocyte clone (≥ 2 RFP^–^ cardiomyocytes) in the sham hearts, but the number of RFP^–^ cardiomyocyte clones was significantly increased in the PAB group (Figure 3C). These data further support that pressure overload can also induce cardiomyocyte proliferation.

### There Was No M2 Macrophage Accumulation in P7 PAB Hearts

**FIGURE 4:**
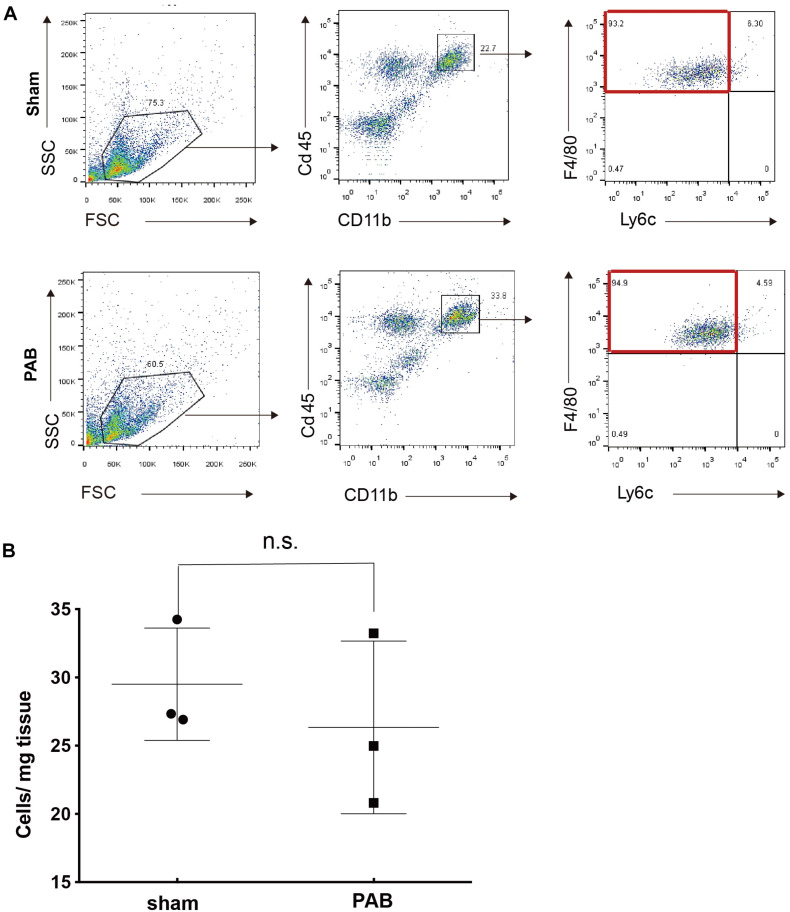
No significant accumulation of M2 marophages in P7 PAB groups hearts. **(A)** FACS gating scheme for the identification of CD45^+^CD11b^+^F4/80^+^Ly6c^–^ M2 macrophage. **(B)** Quantification of M2 macrophage as the cell number per mg of tissue (*n* = 3). Data are presented as Mean ± SEM, Statistic analysis was performed with two-tailed Student’s *t*-test. n.s., non-significant.

Anti-inflammatory F4/80^hi^Ly6c^lo^ M2 macrophage is an essential mechanism required for myocardial regeneration of neonatal heart after mechanic or ischemic injuries (Aurora et al., 2014). At these pathological conditions, this type of anti-inflammatory M2 macrophage accumulates in injured heart and contributes to cardiomyocyte proliferation. We wondered whether M2 macrophages also have a role in facilitating cardiomyocyte proliferation in neonatal PAB heart. We collected sham and PAB hearts at P7 for FACS analysis. Results showed that the dominant type of Cd45^+^Cd11b^+^ myeloid cells in both sham and PAB hearts is F4/80^hi^Ly6c^lo^, the same features as the previously reported cardiac resident macrophage (Aurora et al., 2014). However, there was no significant difference in the number of M2 macrophages between the sham and PAB hearts (Figures 4A,B). These results indicate that the proliferation of neonatal cardiomyocyte could occur independent of M2 macrophage accumulation. The capability of proliferation is more of an inherent characteristic of neonatal cardiomyocyte.

### Gene Expression Changes in Response to Neonatal PAB

**FIGURE 5:**
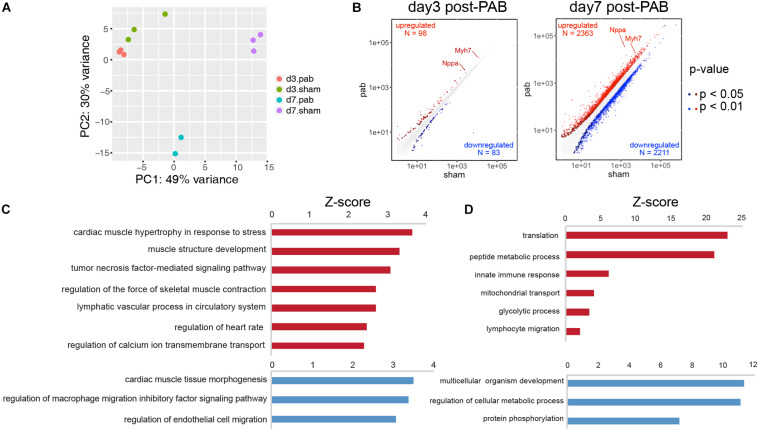
Transcriptome analysis of PAB hearts. **(A)** Principal component analysis of transcriptional profiles of PAB and sham samples. **(B)** Scatter plots showed DEGs in P3 and P7 PAB hearts. **(C,D)** Gene ontology analysis of DEGs at P3 **(C)** and P7 **(D)**.

To investigate the molecular mechanisms of cardiomyocyte hyperplasia and hypertrophy in response to the pressure overload, we performed PAB on P0 mice and harvested ventricles 3 and 7 days after the surgery for RNA-seq. Age-matching sham operated mice were used as control groups. The principal component analysis (PCA) revealed dominant factors that contribute to transcriptional variance among different conditions. The first principal components (PC) was mostly affected by the age of the animals, while the second PC was subject to the influence of PAB operation (Figure 5A). Differential gene expression analysis showed that only 98 genes were up-regulated and 83 down-regulated in PAB hearts at P3, compared to 2,363 up-regulated and 2,211 downregulated in P7 PAB hearts (Figure 5B), suggesting an accumulative buildup of transcriptional changes. Among them, the hypertrophy marker genes, such as *Nppa* and *Myh7*, were highly responsive to pressure overload and were among the top up-regulated genes even at early stage (Figure 5B). To further determine the molecular and cellular functions of differentially expressed genes (DEG), we performed gene ontology analysis. Although there were only a few DEGs in P3 PAB hearts (Figure 5C), they were enriched in functions such as cardiac muscle hypertrophy, muscle contraction, and ion transport, suggesting that hypertrophy was a sensitive and major response induced by pressure overload, which occurred prior to obvious phenotypic change. In P7 PAB hearts, up-regulated DEGs were highly involved in translation, metabolic process and mitochondrial activities (Figure 5D), indicating robust protein synthesis and high-level energy metabolism.

### PAB Had Differential Impacts on Various CM Programs

**FIGURE 6:**
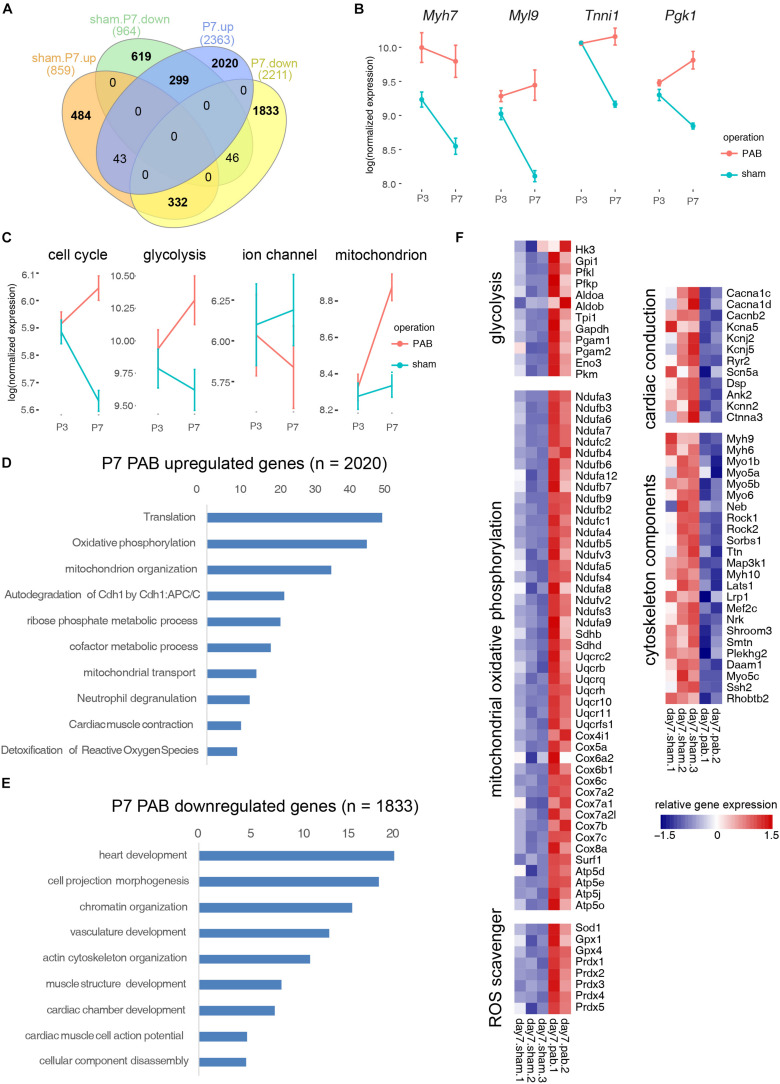
Functional categorization of DEGs induced by cardiac maturation and pressure overload. **(A)** Venn diagram showing overlay between DEGs during postnatal cardiac maturation and DEGs induced by PAB. **(B)** Examplary fetal gene expression changes. **(C)** Expression dynamics of CM programs. **(D)** Gene ontology analysis of PAB-specific up-regulated genes. **(E)** Gene ontology analysis of PAB-specific down-regulated genes. **(F)** Heatmap showing functional gene groups involved in pressure overload response.

CM programs include improved organization of sarcomeric structures, sarcomeric isoform switching, expressing higher levels of ion channels, switching main energy source from glycolysis to β-oxidation, and most significantly, cell cycle exit. To dissect how individual CM programs respond and interconnect under pressure overload, we compared the transcriptomes of P3 and P7 sham hearts and overlaid the resultant DEGs with DEGs derived from comparing the transcriptome of P7 sham and PAB hearts. We found that 299 genes were up-regulated in P7 PAB hearts and down-regulated during day 3–7 postnatal development (Figure 6A), including fetal-specific sarcomeric isoforms (*Myh7*, *Tnni1*, and *Myl9*) and a glycolysis gene encoding phosphoglycerate kinase (*Pgk1*) (Figure 6B). The retention of fetal-specific gene expression suggests a delayed CM progress. On the other hand, 332 genes were down-regulated in P7 PAB hearts and up-regulated in P7 sham hearts compared to P3 sham hearts (Figure 6A). In total, the pressure overload-induced DEGs overlaid over 30% DEGs occurring during postnatal cardiac growth, but in opposite direction. It suggests a disrupted CM progress.

To assess the gene expression change of individual CM program, we categorized genes into functional modules and measured the average gene expression for each signature CM program such as cell cycle, glycolysis, ion channel, and mitochondrial oxidative phosphorylation. Interestingly, we noted that different CM programs responded differently to pressure overload. The overall gene expression in cell cycle and glycolytic metabolism decreased during normal postnatal development, however they increased in the PAB hearts along the time (Figure 6C). In contrast, ion channel gene expression increased postnatally, but reduced in the PAB group (Figure 6C). These expression profile changes suggest that pressure overload deterred neonatal cardiomyocyte maturation. On the other hand, the mitochondrial component genes, which normally increased with the enhanced mitochondrial biogenesis in postnatal cardiomyocyte, was further up-regulated under pressure overload, suggesting elevated energy demand (Figure 6C).

To identify PAB-specific pathological responses, we performed gene ontology analysis on genes only significantly expressed in P7 PAB hearts. Results showed that genes participating in translation, oxidative phosphorylation, reactive oxygen species (ROS) scavenger programs were up-regulated (Figures 6D,F), while cytoskeletal genes were down-regulated under pressure overload (Figures 6E,F). These results suggested that specific effects of pressure overload on neonatal cardiomyocytes are to promote protein synthesis and energy production through mitochondria, which may contribute to cardiac hypertrophic growth. Interestingly, pressure overload also triggered a specific gene program—ROS scavengers, which might protect the heart from tissue damage and severe fibrosis in short term.

## Discussion

Postnatal cardiac growth is characterized by cardiomyocyte hypertrophy. Rodent cardiomyocytes quickly become binucleated or multi-nucleated shortly after birth (Li et al., 1996). Our data indicate that PAB further promotes cardiomyocyte hypertrophy and the polyploidization and multinucleation of cardiomyocytes are accelerated under increased afterload. It indicates that normal neonatal hearts also use cellular hypertrophy, the adult cardiac response to pressure overload, as a compensatory mechanism to ensure steady hemodynamics. Disproportionated cardiac hypertrophy is often seen in the early postnatal development of preterm infants so that the reduced cardiac mass relative to body size at birth are quickly normalized after early postnatal development (Aye et al., 2017). In a mouse model with impaired intrauterine cardiac development, underdeveloped hearts also restore normal heart size after birth through enhanced cardiomyocyte hypertrophy (Drenckhahn et al., 2015). Taken together, these results indicate that neonatal hearts can respond to stress through cardiomyocyte hypertrophy.

Numerous studies have shown that neonatal hearts, unlike adult ones, have a strong proliferative capability to regenerate lost cardiomyocytes after physical or ischemic injuries (Lam and Sadek, 2018). Our data and others indicate that neonatal hearts also have enhanced cardiomyocyte proliferation in response to pressure overload (Malek Mohammadi et al., 2019). Interestingly, there is no accumulation of M2 macrophages in P7 PAB hearts, although robust cardiomyocyte proliferation has occurred at this time point. It is unlike other neonatal injury model, in which increased M2 macrophages are required for heart regeneration (Aurora et al., 2014). It suggests that proliferation under pathological condition could be an autonomous response of neonatal cardiomyocytes. It is more of intrinsic property of neonatal cardiomyocytes. Although we didn’t detect significantly increased M2 Macrophages in P7 PAB hearts, RNA-seq analysis has shown that expression of genes involving innate immune response and lymphocyte migration are up-regulated in P7 PAB hearts, suggesting an innate immune response. Previous studies have shown that adult transverse aortic constriction (TAC) transiently increases proinflammatory (M1) macrophage infiltration but not reparative (M2) macrophages in P7 TAC hearts (Patel et al., 2017, 2018), which is consistent with our observation. qPCR assays indicate that adult TAC produces a proinflammatory cardiac milieu characterized by upregulated expression of *Ccl2*, *Tnf*, and *Il-4* (Patel et al., 2018). Although we note increased expression of *Ccl2* in neonatal PAB hearts, there is no change in the expression of *Tnf* and *Il-4*. It is worth exploring whether neonatal PAB also induces proinflammatory response and T cell expansion in the future study.

Cardiomyocyte is a single cell type, but this type of cells is heterogenous in many aspect (Cui et al., 2020). Cycling cardiomyocytes in adult hearts are composed of hypoxic mononucleated cardiomyocytes with a characteristic of neonatal cardiomyocytes (Kimura et al., 2015). A newly identified neonatal cardiomyocyte subpopulation with high proliferative capability is more immature than its counterparts at the same stage (Cui et al., 2020). In a similar study, neonatal cardiomyocytes can be divided into two distinct populations according to *Tnnt2* expression levels (Martinez et al., 2019). Immature cardiomyocytes with low *Tnnt2* expression level present high mitotic activities and conversely, mature cardiomyocytes with high *Tnnt2* expression level usually have less mitotic activities. When we were testing *Tnnt2* promoter-driven Tet-on lineage-tracing tool, we noticed that a very low frequency of cardiomyocytes was not labeled by this tool, even when we induced Cre expression at late gestation stage. Taking advantage of this rare event, we observed the clonal expansion of cardiomyocytes in hearts under pressure overload. Since non-cardiomyocytes and cardiomyocytes are separated at mid-gestation stage and there is no contribution of non-cardiomyocyte lineage into cardiomyocyte lineage ever since (Li et al., 2019), the unlabeled cardiomyocyte clones should not be derived from cardiac progenitors after doxycycline induction. Rather, it is more likely due to the expansion of existing proliferative cardiomyocytes. As aforementioned, immature neonatal cardiomyocytes have higher proliferative capability (Cui et al., 2020; Martinez et al., 2019). The unlabeled cardiomyocytes in our lineage-tracing system may represent the immature population with low *Tnnt2* expression environment, in which *Tnnt2* promoter is not sufficient to induce Cre-mediated recombination, but with higher proliferation capability that enables a clonal expansion.

Cardiomyocyte maturity reversely correlates with its proliferative capability. It is one of reasons why adult heart loses the regenerative capability. In regenerating cardiomyocytes, including neonatal mammalian hearts, zebrafish heart regeneration, and genetic modified regenerative adult mouse hearts, the re-entry of cell cycle of cardiomyocytes is often associated with the reverse process of maturation (Lam and Sadek, 2018). Factors affecting cardiomyocyte maturation often promote cardiomyocyte proliferation. Cardiac myosin binding protein C (Mybpc3) is a structural component of cardiac sarcomere that affects myofibril integrity and contractility. *Mybpc3* knockout mice develop cardiomegaly by postnatal day 9, while the heart size is normal at birth. The rapid mass growth at the neonatal stage is predominantly mediated by cardiac hyperplasia rather than hypertrophy (Farrell et al., 2017). Metabolic shift from glycolysis to β-oxidation is a major cellular change during neonatal cardiomyocyte maturation to adapt the normoxic environment and the change in energy substrate availability (Bailleul et al., 2020). Increasing glucose uptake in neonatal heart could significantly enhance post-injury regeneration and cardiomyocyte proliferation (Martinez et al., 2019). On the contrary, inhibition of fatty-acid utilization significantly prolongates the proliferative window of neonatal cardiomyoctes (Cardoso et al., 2020). Our transcriptomic analysis indicates that gene expression changes induced by pressure overload counteracts some of CM programs, such as inhibition of glycolytic metabolism, up-regulation of cardiac ion channel gene expression and down-regulation of fetal-specific sarcomeric isoforms. The maintenance of immature cardiomyocyte status may contribute to the enhanced cardiomyocyte proliferation in PAB hearts. Meanwhile, the highly activated translation machinery as well as enhanced mitochondrial biogenesis and function programs under pressure overload are likely the mechanisms promoting cardiomyocyte hypertrophy. It will be interesting to dissect whether the expression alterations with opposite effects are due to discrepant responses of different cardiomyocyte subpopulations or the bivalent expression changes occur in the majority of neonatal cardiomyocytes.

## Data Availability Statement

RNA-seq data have been deposited in NCBI’s Gene Expression Omnibus (GSE159969). All codes used in the manuscript for RNA-seq analyses are available upon request.

## Ethics Statement

The animal study was reviewed and approved by the Animal Care and Use Committee of Shanghai Children’s Medical Center.

## Author Contributions

XD, SW, and YW performed the experiments. JY did mouse genotyping and husbandry. ZZ, JL, and NB designed the studies. XD, SW, and ZZ analyzed the data and wrote the manuscript. JL and NB edited the manuscript. All authors contributed to the article and approved the submitted version.

## Conflict of Interest

The authors declare that the research was conducted in the absence of any commercial or financial relationships that could be construed as a potential conflict of interest.

## Footnotes

1 http://metascape.org

## Supplementary Material

Supplementary Figure S1 Cardiomyocyte apoptosis in P7 PAB hearts. **(A)** Representative confocal images for TUNEL (green), cTnT (red) and DAPI (blue) staining. Arrowheads, apoptotic cardiomyocyte. **(B)** Quantification of TUNEL + cardiomyocytes (*n* = 4). Data are presented as Mean ± SEM. Two-tailed Student’s *t*-test. n.s, non-significant. Scale bar: 25 μm.
