# Supplementary material for: Neonatal Heart Responds to Pressure Overload With Differential Alterations in Various Cardiomyocyte Maturation Programs That Accommodate Simultaneous Hypertrophy and Hyperplasia
Source: Front Cell Dev Biol. 2020 Nov 19;8:596960. doi: 10.3389/fcell.2020.596960 (PMC7710899; doi:10.3389/fcell.2020.596960)
Supplement: Supplementary Figure S1 — Cardiomyocyte apoptosis in P7 PAB hearts. (A) Representative confocal images for TUNEL (green), cTnT (red) and DAPI (blue) staining. Arrowheads, apoptotic cardiomyocyte. (B) Quantification of TUNEL + cardiomyocytes (n = 4). Data are presented as Mean ± SEM. Two-tailed Student’s t-test. n.s, non-significant. Scale bar: 25 μm. [file Image_1.pdf]

Supplementary materials for

Neonatal heart responds to pressure overload with differential alterations in various cardiomyocyte maturation programs that accommodate simultaneous hypertrophy and hyperplasia

Xiaoning Ding[1, *], Shoubao Wang[2, 3, *], Ye Wang[1], Junjie Yang[1], Nan Bao[4, #], Jinfen Liu[2, #], Zhen Zhang[1, #]

[1]Pediatric Translational Medicine Institute and Shanghai Pediatric Congenital Heart Disease Institute, Shanghai Children's Medical Center, Shanghai Jiao Tong University School of Medicine, Shanghai 200127, China.

[2]Department of Thoracic and Cardiovascular Surgery, Shanghai Children's Medical Center, Shanghai Jiao Tong University School of Medicine, Shanghai 200127, China.

[3]Present address: Department of Plastic and Reconstructive Surgery, Shanghai Ninth People's Hospital, Shanghai Jiao Tong University School of Medicine, Shanghai 200011, China.

[4]Department of Pediatric Surgery, Shanghai Children's Medical Center, Shanghai Jiao Tong University School of Medicine, Shanghai 200127, China.

*These authors contributed equally.

#Correspondence to: Nan Bao, bnscmc@shsmu.edu.cn; Jinfen Liu, liujinfen2002@126.com ; Zhen Zhang, zhenzhang@sjtu.edu.cn.

Supplementary Figure

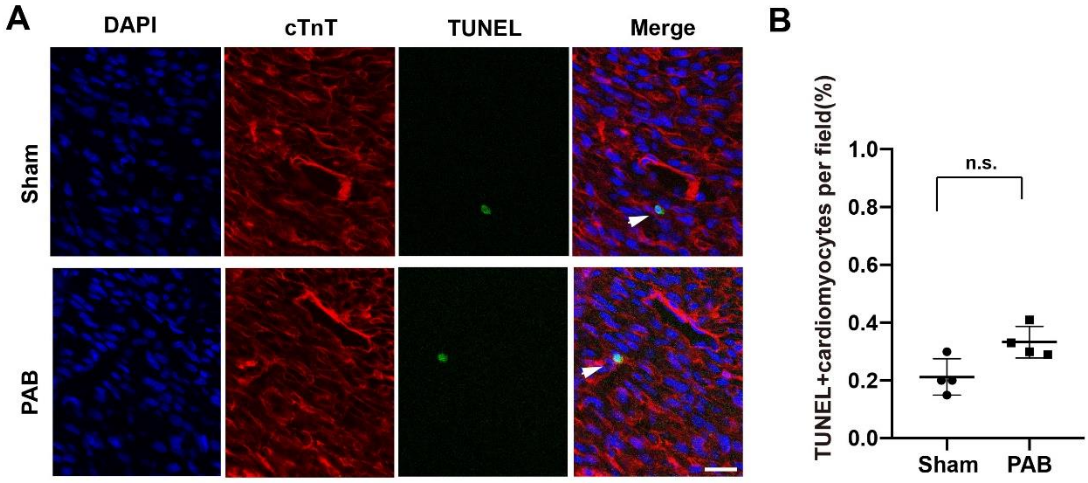

Figure S1. Cardiomyocyte apoptosis in P7 PAB hearts. **(A)** Representative confocal images for TUNEL (green), cTnT (red) and DAPI (blue) staining. Arrowheads, apoptotic cardiomyocyte. **(B)** Quantification of TUNEL$^+$ cardiomyocytes (n=4). Data are presented as Mean ± SEM. Two-tailed Student's t test. n.s, non-significant. Scale bar: 25μm.
